# LncRNA: A Potential Target for Host-Directed Therapy of *Candida* Infection

**DOI:** 10.3390/pharmaceutics14030621

**Published:** 2022-03-11

**Authors:** Ye Wang, Hongdan Xu, Na Chen, Jin Yang, Hongmei Zhou

**Affiliations:** State Key Laboratory of Oral Diseases, National Center of Stomatology, National Clinical Research Center for Oral Diseases, Frontier Innovation Center for Dental Medicine Plus, West China Hospital of Stomatology, Sichuan University, Chengdu 610041, China; 2020324035087@stu.scu.edu.cn (Y.W.); xuhongdan@stu.scu.edu.cn (H.X.); chenna@stu.scu.edu.cn (N.C.)

**Keywords:** *Candida*, *Candida*–host interaction, host-directed therapy (HDT), lncRNA

## Abstract

Despite various drugs work against *Candida*, candidiasis represents clinical management challenges worldwide due to the rising incidence and recurrence rate, as well as epidemics, of new drug-resistant pathogens. Recent insights into interactions between *Candida* and hosts contribute to exploring novel therapeutic strategies, termed host-directed therapies (HDTs). HDTs are viable adjuncts with good efficacy for the existing standard antifungal regimens. However, HDTs induce other response unintendedly, thus requiring molecular targets with highly specificity. Long noncoding RNAs (lncRNAs) with highly specific expression patterns could affect biological processes, including the immune response. Herein, this review will summarize recent advances of HDTs based on the *Candida*–host interaction. Especially, the findings and application strategies of lncRNAs related to the host response are emphasized. We propose it is feasible to target lncRNAs to modulate the host defense during *Candida* infection, which provides a new perspective in identifying options of HDTs for candidiasis.

## 1. Introduction

Candidiasis is one of the most common opportunistic fungal infections, ranging from superficial mucocutaneous infections to systemic infections [1,2]. Major risk factors for *Candida* infection include compromised immune systems, a history of major surgery (particularly abdominal surgery with intestinal fluid leakages), and treatment with glucocorticosteroids or broad-spectrum antibiotics [3,4]. Superficial infections are easily curable; however, invasive candidiasis is widely accepted as a significantly life-threatening infection affecting about 400,000 persons per year [5], and its incidence rate is on the rise [6,7]. Lately, *Candida* secondary infections following COVID-19 disease have been widely reported, and candidiasis leads to elevated risks of mortality [8]. Candidiasis is also a huge economic burden on society and on the patient’s family, as each hospitalized patient with invasive candidiasis costs around 46,684 dollars in the United States [9]. In addition, *Candida* infection has been implicated as a risk factor for tumorigenesis and tumor progression [10,11,12]. Therefore, *Candida* infection is a significant public health concern at present.

Conventional antifungal drugs used in candidiasis include azoles, allylamines, morpholines, polyenes, echinocandins, chitin synthase inhibitors, and fluorinated pyrimidine analogs [13], which target the pathogen components directly. However, their efficacy has recently been challenged due to the off-target host toxicity, lower bioavailability, and emerging drug resistance [14,15]. Notably, the abuse of antifungal drugs also promotes the occurrence of new pathogens in clinical settings [15]. For instance, *Candida Auris* is an emerging multidrug-resistant nosocomial pathogen and associated with invasive candidiasis and high mortality rates [16,17]. Given the issues of treatment with conventional antifungals, there is a consensus on the urgent need for novel targeted and effective therapeutic strategies.

As the predisposing factors of fungal diseases mainly reside in weakened immune system, a new strategy named host-directed therapies (HDTs) is becoming a viable adjunct to existing antifungal regimens. HDTs may be used to cure relapsing infections or develop drugs against emerging fungi. The current therapies are focusing on the protection of the innate immune response and the control of the adaptive immune response, including cytokine therapy, cellular immunotherapy, vaccine, and antibody therapy. Nevertheless, several challenging concerns limit the exploration of HDTs. Since the accurate and effective implementation of HDTs is built on knowledge of the host–*Candida* interaction, the development of molecular medicine brings a promising light to identifying potential targets for intervention. Long noncoding RNAs (lncRNAs) have been considered to play important roles in almost all the biological processes [18]. Although the effect of lncRNAs on candidiasis remain largely unknown, numerous studies have revealed that lncRNAs participate in other pathogen-infected diseases through regulating similar immune responses. This review is intended to summarize the host response and relevant host-directed application strategies in candidiasis, especially those focused on recent advances and clinical studies of lncRNAs related to the host defense, paving the way for lncRNA-targeted HDTs for *Candida* infection.

## 2. Interactions between *Candida* and Host

*Candida* is a major opportunistic pathogen. On the one hand, the virulence of *Candida* is associated with the morphological transition between yeast and hyphal forms, and switching contributes to the capacity of penetrating and other virulence traits [19]. Virulence factors of *Candida*, such as adhesins, invasins, and secreted hydrolases, destroy the physical barriers of the skin and mucosa to create channels for invasion [20,21]. Meanwhile, the pathogen-associated molecular patterns (PAMPs), represented by glucan and mannan, are recognized by pattern recognition receptors (PRRs) on host cells [21]. On the other hand, the host response to candidiasis requires a chain of process, including the recognition of fungal, the activation of related immune signal cascades, and the ultimate clearance of pathogens [22,23]. Various cells such as epithelial cells, fibroblasts, monocytes, neutrophils, dendritic cells (DCs), and T and B lymphocytes, as well as multiple soluble molecules and cytokines, are enrolled in the process. 

### 2.1. Recognition of Candida by Pattern Recognition Receptors

**Figure 1:**
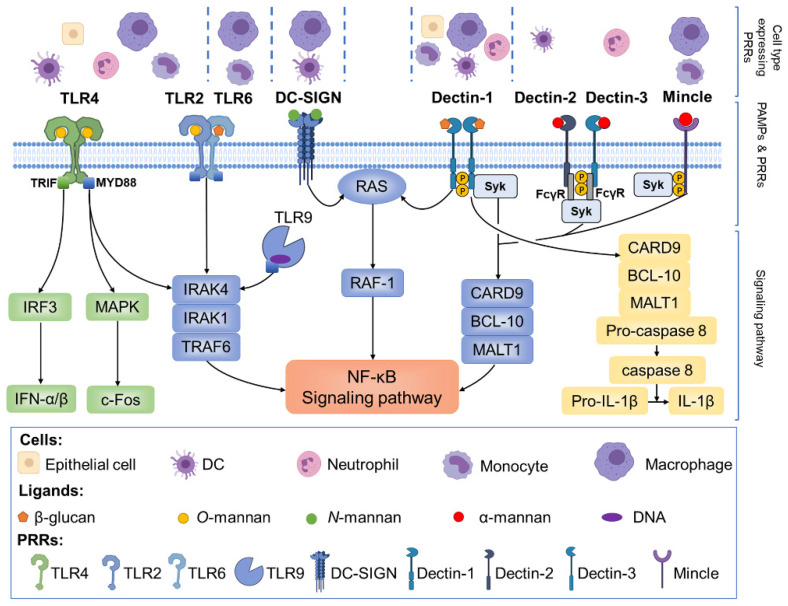
The main PRRs involved in immunity to *Candida*. Cell surface TLR2, TLR4, and TLR6 sense mannans and intracellular TLR9 sense DNA, and they all signal to IRAKs through MYD88. Then, the phosphorylation of IRAK4/IRAK1/TRAF6 activates the NF-κB pathway. Dectin-1 and DC-SIGN also activate NF-κB signaling via the RAS-RAF-1 pathway. Moreover, the dectin-1 homodimer, the dectin-2 and dectin-3 heterodimers, and Mincle recruit Syk in a FcγR-dependent manner to form a CARD9/BCL-10/MALT1 scaffold, which activates NF-κB in a classical way. In addition, dectin-1 mediates the formation of the CARD9/BCL-10/MALT1 complex to promote IL-1β secretion via the non-classical caspase-8 pathway. In response to TLR4, the MAPK/c-Fos pathway is also activated by MYD88, while the transcription of IFN-α/β is promoted by TRIF-mediated IRF3. PRRs, pattern recognition receptors; TLR, toll-like receptor; IRAKs, the interleukin-1 receptor-associated kinases; MYD88, myeloid differentiation primary response 88; IRAK4, interleukin-1 receptor-associated kinase 4; IRAK1, interleukin-1 receptor-associated kinase 1; TRAF6, tumor necrosis factor receptor-associated factor 6; NF-κB, the nuclear factor-kappa B; DC-SIGN, dendritic cell-specific intercellular adhesion molecule-3-grabbing non-integrin; Mincle, macrophage-inducible C-type lectin; FcγR, Fc-gamma receptor; CARD9, caspase recruitment domain containing protein 9; BCL-10, B-cell lymphoma 10; IL, interleukin; MAPK, mitogen-activated protein kinases; IFN, interferons; TRIF, Toll/IL-1R domain-containing adaptor-inducing IFN-beta; IRF3, interferons regulatory factor 3.

The PRRs can be divided into four major classes: Toll-like receptors (TLRs), C-type lectin receptors (CLRs), nucleotide-binding domain leucine-rich repeat-containing receptors, and retinoic acid-inducible gene-I receptors [24]. Synergistic interactions among different PRRs initiate the downstream immune response against *Candida* and induce the production of cytokines and antimicrobial peptides (AMPs) [25,26]. Among them, it is mainly TLR- or CLR-induced cascade signaling that responds to *Candida* (Figure 1). 

The main PAMPs of s *Candida* cell wall include β-glucan, simple linear chain *O*-mannan, and *N*-mannan with multiple lateral α- or β-mannose branches. TLR2, TLR4, and TLR6 are cell membrane-associated, whereas TLR9 is located inside the cells [25]. TLR2 and TLR4 recognize *O*-mannan, TLR6 is involved in the recognition of β-glucan, and TLR9 detects fungal DNA [27,28]. The TLR2–TLR6 heterodimers, together with cell surface TLR4 dimers and intracellular TLR9 [29,30], are reported to signal to the interleukin (IL)-1 receptor-associated kinases (IRAKs) through adaptor protein MYD88, thus forming a complex with IRAK4, IRAK1, and TNF receptor-associated factor 6 (TRAF6) [31,32]. nuclear factor-kappa B (NF-κB) can be activated by TRAF6-induced cascade signaling to initiate the transcription process of inflammatory cytokines (tumor necrosis factor (TNF), IL-1, and IL-6) and chemokines (IL-8, CXCL1, CCL3, and CXCL2) [33]. In addition, another signaling pathway induced by TLRs also plays a role in the antifungal response. The mitogen-activated protein kinase (MAPK) signaling pathway can be activated by TLR4, increasing c-Fos expression [34], contributing to the clearance of infected cells [35,36]. TLR4 also activates the *IFNG* gene via IFN regulatory factor 3 (IRF3) [37,38]. As another type of PRR, CLRs include dectin-1, dectin-2, dectin-3 [39], macrophage-inducible C-type lectin (Mincle), and dendritic cell specific intercellular adhesion molecule-3-grabbing non-integrin (DC-SIGN) [24]. They mainly recognize β-glucan, α-mannan, and *N*-mannan [26]. After combining with the ligands, the dectin-1-dectin-1 dimers, dectin-2-dectin-3 dimers, and Mincle can recruit Syk in a FcγR-dependent manner to form a CARD9/BCL-10/MALT1 scaffold, which activates the nuclear translocation of NF-κB in a classical way [39,40]. Syk/CARD9 has been demonstrated as crucial signaling involved in mediating protection against different *Candida* species [41]. NF-κB can be activated in a different way by dectin-1 and DC-SIGN through the RAS-RAF-1 pathway [39,42]. In addition, dectin-1 can promote inflammation against *Candida* by activation of the inflammasome, such as the NOD-like receptor protein 3 (NLRP3) inflammasome [43,44]. Taken together, the NF-κB pathway could be activated by both TLR- and CLR-induced cascade signaling and is critical downstream after PRR recognition.

### 2.2. Innate Immunity against Candida 

**Figure 2:**
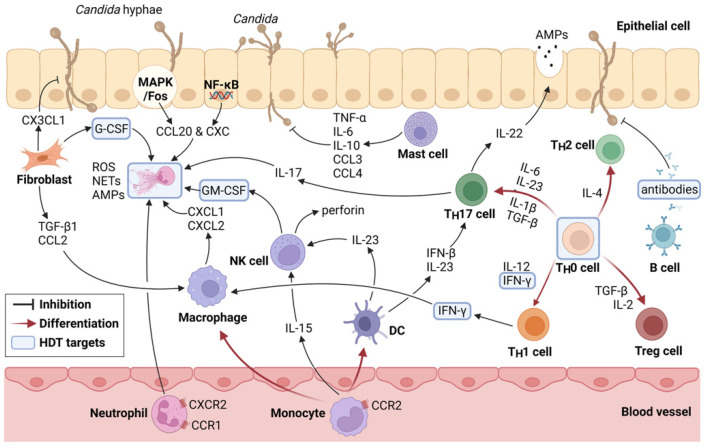
Mechanisms against *Candida* involving innate and adaptive immunity. Host cells play an anti-*Candida* role directly by secreting cytokines with antifungal activities, such as AMPs secreted by epithelial cells, CX3CL1 secreted by fibroblasts, and IL-6 secreted by MCs. In addition, antifungal cytokines also include perforin secreted by NK cells, IL-22 produced by T_H_17 cells, antibodies produced by B cells, etc. Additionally, host cells play the antifungal role indirectly by the recruitment of immune cells or via affecting the differentiation and activation of immune cells. Some cells and cytokines mentioned above have become targets of the current HDTs of *Candida*. AMPs, antimicrobial peptides; CX3CL1, C-X3-C motif chemokine ligand 1; TGF-β1, transforming growth factor beta1; G-CSF, granulocyte-colony stimulating factor; CCL, C-C motif chemokine ligand; MCs, mast cells; TNF-α, tumor necrosis factor alpha; CXCR, C-X-C chemokine receptors; CCR, CC Chemokine receptor; GM-CSF, granulocyte macrophage colony-stimulating factor; CXCL, C-X-C motif ligand; NK cells, natural killer cells; ROS, reactive oxygen species; NETs, neutrophil extracellular traps; HDT, host-directed therapy.

After the initial recognition and PRRs-mediated cascades, the innate immune response is initiated [45]. The innate immune response requires neutrophils, monocytes, and other cell types, including epithelia and fibroblasts (Figure 2). 

The innate cells include neutrophils, monocytes, macrophages, natural killer (NK) cells, etc. They mainly undergo two stages of recruitment and functional activation. We propose that, among them, monocytes are the key points of the innate immune network against *Candida* infection. Neutrophils are the main effector cells in the early stage of infection [2]. Neutrophils in the blood are recruited to the infected site by CXCR2 (early stage of inflammation)- and CCR1 (late stage)-stimulated chemotaxis [46,47]. IL-17 secreted by T_H_17 cells and granulocyte-macrophage colony-stimulating factor (GM-CSF) secreted by NK cells also induce the recruitment and activation of neutrophils. Of note, neutrophils could be recruited and activated by monocytes/macrophages: tissue-resident macrophages mediate endocytosis and secrete CXCL1 and CXCL2 at the infected site to act on neutrophils [48,49,50]. The recruited neutrophils can utilize phagocytosis, reactive oxygen species (ROS), and neutrophil extracellular traps (NETs) to capture and kill the invading pathogens [51,52]. Neutrophils remove pathogens mainly through phagocytosis. Phagocytosis is amplified by leukotriene B4, which are induced by hyphae specifically and require ROS [53]. Extracellular pathogens are eliminated by relying on NETs and degranulation [54]. Then, in the later stage of systemic infection, monocytes play a more critical role [55]. Monocytes in the blood are recruited to the infected site by CCR2-stimulated chemotaxis [56]. When monocytes are stimulated and activated by PAMPs, glucose metabolism associated with ATP production is altered from aerobic oxidation to glycolysis. Recent studies have revealed that metabolic reprogramming after *Candida* stimulation affects the immune functions of monocytes [57]. Monocytes can differentiate into macrophages (M1, classically activated macrophages) to activate the immune defense. Mechanistically, dectin-1 and TLR2 expressed on macrophages induce activation of the NF-κB-signaling pathway or upregulate the inflammation response through NLRP3 inflammasome activation [58]. However, macrophages could also turn into anti-inflammatory phenotypes (M2, alternatively activated macrophages) to activate immune escape [59]. Macrophages destroy *Candida* mainly by nutrient deprivation, low pH, and oxidative stress in the phagosome [60]. *Candida* can form lengthy hyphae that are not easy to engulf to escape phagocytosis, while macrophages counteract this by folding the hyphae to help elevate the clearance rate [61]. Monocytes can also differentiate into DCs. DCs work as the link between innate immunity and the adaptive immunity. After phagocytosis of DCs, the antigens are processed into antigenic peptides and presented to T lymphocytes [62]. Mechanistically, DCs can secrete IL-23 or IFN-β to activate T_H_17 cells [63,64]. Furthermore, IL-23 and IL-15 secreted by monocytes can activate NK cells that can eliminate the target fungus either by direct cytotoxic abilities or by death receptor-mediated apoptosis [50,65]. They also release chemokines and cytokines such as GM-CSF to modulate the activity of other immune cells [49,66]. Therefore, because of the extensive effects of monocytes on neutrophils, macrophages, DCs, and NK cells, monocytes become an important node in the functional reprogramming of the innate immunity induced by *Candida*.

There is new evidence suggesting that other cells are also involved in fungal infection, including epithelial cells, fibroblasts, and mast cells (MCs). Epithelial cells, fibroblasts, and amounts of the extracellular matrix are the main components of the skin and mucosa, which are the first physical barrier of the host defense. Epithelial cells and fibroblasts are regarded as ‘noncanonical immune cells’ and ‘a non-classical type of the innate immune system’, respectively [67,68]. Epithelial cells are responsible for detecting, responding, and killing *Candida* spp. Epithelial cells monitor the microbiome composition and the expression of microRNAs in epithelial cells varied with high or low pathogenic *Candida* species [68]. The MAPK1 and Fos signaling pathways in epithelial cells can be activated by *Candida* hyphae, while NF-κB-signaling pathways can be activated in a TLR4-dependent way by both *Candida* hyphae and yeast [69]. The release of IL-1α upon candidalysin exposure and its ligand epidermal growth factor receptor could also play a synergistic role in initiating an early innate epithelial response [70]. Under the stimulation of these signals, epithelial cells may secrete AMPs, including β-defensins, cathelicidin LL-37, and S-100 protein [71], suggesting epithelial cells exert a direct antifungal effect. Epithelial cells can also recruit neutrophils and monocytes to the infectious site via chemokines CXC and CCL20, [72] thus mediating the antifungal effect indirectly. As important mesenchymal cells in the physical barrier, fibroblasts could acquire secretory functions in response to tissue injury. Our preliminary studies reported that fibroblasts could secrete CX3CL1 to effectively prevent the adhesion and invasion of *Candida* [73]. Fibroblasts may also play indirect antifungal roles by recruiting and altering the functional status of immune cells through transforming growth factor-β1 (TGF-β1), granulocyte colony-stimulating factor (G-CSF), or CCL2 [74,75]. In addition, the crosstalk between fibroblasts and immune cells was reported in cancer [76]. MCs mainly residing in tissues exposed to the environment are one of the first cell lines in the immune system to interact with invading pathogens. MCs can be activated by dectin-1 and TLR2 and then kill *Candida* through degranulation; phagocytosis; the production of NO; and release of cytokines such as TNF-α, IL-6, IL-10, CCL3, and CCL4 [77,78,79]. Like macrophages that can prevent unnecessary tissue damage caused by overreaction of the immune system through differentiation into M2, MCs have similar immunomodulatory functions. *Candida*-infected MCs promote macrophage chemotaxis, while resting MCs inhibit macrophage phagocytosis [80]. It has been reported that MCs, together with IL-9, are key players of *C. albicans* commensalism and pathogenesis [81]. These studies revealed that MCs play a role in maintaining the delicate balance between host immunity and the microbiota. Therefore, epithelium cells, fibroblasts, and MCs in the host’s first barrier both perform antifungal functions via secreting antimicrobial factors directly or initiating the immune response against *Candida* infections indirectly.

### 2.3. Adaptive Immunity during Candida Infection

Adaptive immunity can be divided into T-cell-mediated cellular immunity and B-cell-mediated humoral immunity. It is well-known that the adaptive immune response to *Candida* infections is mainly mediated by different subsets of CD4^+^ T (T_H_) lymphocytes. For instance, in AIDS patients, the T_H_ cell count is depleted and the prevalence of candidiasis increased [82]. There are mainly four different subtypes of T_H_ cells (T_H_1, T_H_2, T_H_17, and Treg). T_H_1 cells are induced by DCs via IL-12 when exposed to *Candida* and secrete IFN-γ to promote the clearance of *Candida*, which causes a positive feedback loop for the DCs to further produce IL-12 and induce more T_H_1 cells [38,83]. T_H_17 cells are mainly induced by IL-6, along with IL-1β and IL-23, and are responsible for clearing pathogens by secreting many cytokines, including IL-17 and IL-22. IL-17 recruits neutrophils to kill *Candida*, and IL-22 can promote the release of β-defensins to enhance the integrity of mucosal epithelium [84]. A recent study revealed that, in order to reduce lung fungal burdens and avoid further complications, platelets activated by candidalysin would release the Wnt antagonist Dickkopf-1, which contributes to T_H_2 and T_H_17 cell polarization [85]. However, the requirement for TGF-β during T_H_17 cell differentiation is age-dependent. In newborns, T cells are biased towards T_H_2 or Treg cell differentiation, causing the increasing risk of susceptibility to candidiasis [86]. Furthermore, Treg cells are essential for maintaining self-tolerance and suppressing the pathological immune response by the clonal deletion of self-reactive T cells during the process of infection [87]. Mechanistically, the formation and differentiation of Tregs are mediated by IL-10, IL-27, and TGF-β [88]. In brief, T_H_1 and T_H_17 immunity contribute to the host defense against *Candida*, while T_H_2 immunity plays an opposite role [89]. 

In contrast, the role of humoral immunity mediated by B cells and their antibodies during *Candida* infection seems less well-characterized. B cells may be activated directly by hyphae via TLR2/MYD88 signaling. Notably, MyD88 signaling is essential for both IgG1 production and IL-6 secretion by B cells, thus promoting T_H_17 polarization [90]. Recent research has reported that B-cell depletion led to reduced T_H_1 and T_H_17 cell responses during *Candida* infection [91]. B cells also produce anti-mannan antibodies, mainly IgM, IgG, and IgA, which can interfere with the germ tube formation of *Candida* [89,92,93]. Antibodies also prevented fungal-mediated epithelial cell adherence and damage by blocking endocytosis [94]. Therefore, the major antifungal mechanism of B cells was to produce antibodies and promote T_H_17 immunity. 

In sum, the innate and adaptive immune cells form an interdependent network. Notably, among the network, the differentiation of monocytes and T_H_ cells plays a key role. They could evolve into several distinct sets that mediate a specific immune response to protect the host against *Candida* infectious challenges (Figure 2). A series of immune-related molecules such as G-CSFs, GM-CSFs, and IFN-γ are involved to destroy the dangerous intruders. Those immune cells and factors involved in the signaling pathway might become potential targets designed for antifungal therapies. 

## 3. Host-Directed Therapies as New Strategies for *Candida* Infections

To beat the formidable opponent, a ubiquitous fungus family, there are two ways: either you cripple your enemy by means of all sorts of antifungals, or you strengthen your defense, relying on individual immunity states. In the past decades, host-directed therapy as an adjunctive strategy focusing on host factors has gained momentum. HDTs in synergy with antifungal therapies aim to enhance the host immunity and promote the clearance of invasive pathogens [95]. As mentioned above, *Candida* infection will mobilize the immune system and cause a host response. The current HDTs mainly enhance the number and/or the function of immune cells or intensify their immune effect, including cytokine therapy, cellular immunotherapy, and vaccine and antibody therapy, which are viable adjuncts to existing antifungal regimens.

### 3.1. Overview of Current HDTs for Candidiasis

At present, cytokine therapy mainly targets G-CSF, GM-CSF, and recombinant IFN-γ (rIFN-γ) [51,96]. G-CSF and GM-CSF mainly affect neutrophils and macrophages [97]. In a randomized placebo-controlled pilot study, combined G-CSF and fluconazole showed a trend for faster infection resolution than fluconazole alone in disseminated candidiasis patients [98], but G-CSF therapy may lead to severe pulmonary complications [99]. In contrast, GM-CSF have been reported to gain complete clinical remission in treating candidiasis with CARD9 deficiency [100]. GM-CSF therapy for refractory mucosal candidiasis showed no adverse events in HIV patients [101,102]. Sargramostim (recombinant human GM-CSF) was also shown to reduce invasive fungal infections after hematopoietic cell transplant [103]. These studies suggested that GM-CSF seems successful as an adjunctive immunotherapy. Eight patients with invasive *Candida* and/or *Aspergillus* infections were treated with rIFN-γ, together with standard antifungal therapy, resulting in partially restored immune function [104]. rIFN-γ immunotherapy in combination with antifungals also reduced the fungal burden in patients with HIV infection [105] and invasive *Candida* infection [96], suggesting that rIFN-γ is a valuable cytokine target.

Another encouraging approach to boosting immunity in patients with candidiasis is cellular immunotherapy, which is based on the re-engineering of immune cells, including neutrophils and T cells. Granulocyte transfusion (GTX) has been used in neutropenic patients to prevent and treat fungal infections for many years. GTX can promote host immunity by restoring the neutrophil count, especially with the emergence of rG-CSF and rGM-CSF [106]. Mario et al. reported two neutropenic patients with life-threatening systemic fungal infections fully recovered after the use of rG-CSF-primed granulocyte transfusions plus amphotericin B [107]. A case–control retrospective analysis suggested that high-dose GTX therapy was associated with better survival rates in high-risk patients with cancer [108]. Cellular immunotherapies target T cells, including adoptive T-cell therapy and chimeric antigen receptor (CAR) T-cell therapy. To date, only one clinical trial has assessed the efficacy of the transfer of *Aspergillus*-specific T cells to patients after haploidentical hematopoietic transplantation, which revealed a good control of mortality caused by fungal infection [109]. The designated CAR T-cells specific for β-1,3-D-glucan were shown to be effective against *Aspergillus fumigatus* in vitro and in vivo using murine models [110], which may be extended to additional fungal pathogens. Recent data detected patterns of T-cell exhaustion in invasive candidiasis patients [111], implying that strengthening the host immunity by checkpoint inhibition is a promising strategy in candidiasis. Although it has not yet been applied to fungal treatments, cellular immunotherapy can also be extended to other cells based on the function mentioned in part 1 and previous research in the cancer field. For instance, Zhao et al. explored a strategy to construct yolk–shell nanohybrids with intrinsic immunomodulatory effects, which can promote the reprogramming of macrophages to the M1 phenotype and induce the maturation of DCs, leading to more effective anticancer therapy [112]. Overall, the cell types targeted by cellular immunotherapy in antifungal therapy and its possible therapeutic effects are worth further exploring. 

Fungal vaccine and antibody therapy is applied to strengthen humoral immunity. Several anti-*Candida* vaccines attempt to utilize live attenuated strains, fungal proteins like the agglutinin-like sequence gene (Als3), cell wall polysaccharides like β-glucan, and glycoconjugates [113]. Most studies remain in the research phase of animal testing; only a few have entered clinical trial stages. For instance, two vaccines containing recombinant *C. albicans*-derived proteins have been shown to be safe and immunogenicity in phase 1/2 clinical trials [114,115]. These fungal vaccines might be applied in patients before organ transplant and those with early HIV infection. The application of antibodies against *Candida* infection seems less well-studied. Monoclonal antibodies against fungal components could inhibit fungal growth and metabolism through opsonization. A double-blind, randomized study revealed that Mycograb, a human recombinant monoclonal antibody against HSP 90, was synergistic with amphotericin B against *Candida* species [116]. Based on the evidence so far, HDT-targeting humoral immunity is a promising alternative approach to preventing and treating candidiasis.

### 3.2. Superiority and Challenge

The discovery of novel antifungals targeting pathogens has gained little progress, as there exist similarities between fungal and human targets [13]. Further complications such as toxicity and drug resistance are also a hurdle to overcome [117]. Obviously, immunotherapy has been taken to the forefront not only in the research field of infectious diseases but in cancers [118]. HDT-based approaches targeting the host rather than pathogens are less prone to traditional antibiotic resistance [119]. To better compare the efficacy level of the approaches above based on evidence, eleven studies were included in this review (Table 1). Among the included eleven articles, six were randomized controlled studies [114,116,120,121,122,123], and five were prospective studies [96,102,124,125,126]. Among the six randomized controlled trials, five articles compared Mycograb [116], NDV-3 [114], NDV-3A [122], anti-CA IgY [120], vitamin D [123], and a placebo, respectively, and the pooled results suggested that the clinical symptoms and incidence of candidiasis in the treatment group were significantly improved. While one article compared different combinations of two drugs, GM-CSF and G-CSF [121], the result showed that, for recipients of allogeneic hematopoietic stem cell transplantation, the prophylactic use of GM-CSF significantly reduced invasive fungal disease-related mortality. Among the five prospective studies, two articles evaluated mucosal candidiasis [102,124], and three articles evaluated invasive candidiasis [96,125,126]. All results suggested that the use of HDTs, especially cytokine therapy, can improve the symptoms of *Candida* infection and reduce the recurrence rate. However, additional clinical evidence is still required for cellular immunotherapy and *Candida* vaccines.

However, despite promising in vitro and in vivo results, the clinical value of HDTs for *Candida*-infected individuals remains to be determined. Until now, most evidence of HDTs for treating candidiasis has been based on small case studies, and larger controlled clinical studies are required to evaluate their efficiency and safety. Besides, both the disease stage and related immune responses are dynamic. Maraviroc, the first-line antagonist for the CCR5 chemokine receptor, has been approved commercially for the treatment of HIV in 2007. However, the agent only works well in HIV patients with early stage who have more CCR5 variants; in contrast, the presence of CXCR4 variants increases with disease progression, causing Maraviroc to fail [127]. This suggests that HDTs should consider the timing of administration and select indications based on more accurate disease typology. Of note, HDTs targeting host factors may cause serious side effects, since they are crucial in many life processes. For example, although IFNs have been widely used in chronic hepatitis, improving the overall efficacy, the side effects are frequent, including flu-like syndromes, fever, fatigue, myelosuppression, alopecia, and injection site reactions [128]. The emergence of anti-programmed cell death 1 ligand antibodies as immune checkpoint inhibitors revolutionized the treatment of cancers, while a large number of patients developed immune-related adverse events marked by end-organ inflammation with T-cell infiltrates [129]. As reported, immunotoxicity and autoimmunity are weaknesses of the current immunotherapeutic approaches [130], and it is conceivable that the side effects will increase in frequency and severity as HDTs become more widespread. We propose a possible improvement by replacing targets, thus reducing the nonspecific inflammatory response. Systems immunology analyses revealed there is consistent overlap between antifungal and antiviral immune responses via integrating diverse transcriptomic studies [43]. These data indicated that molecules targeted by HDTs in the anti-*Candida* host response are also dysregulated in other diseases, thereby requiring molecular targets with highly specificity. The exploration of lncRNAs opens an avenue for the discovery of new specific targets.

## 4. LncRNAs as Potential Therapeutic Targets for HDTs against *Candida*

LncRNAs have been reassessed from initially ‘junk’ transcriptional products to potentially important RNAs. Studies over the past decade have unveiled the important regulatory roles of lncRNAs in various diseases and pointed to the fact that lncRNAs could lead to specific cellular responses and cell fate decisions [131]. Some studies have examined lncRNA profiles following fungal exposure. By high-throughput sequencing analyses, Riege et al. found that lncRNAs in monocytes were differentially expressed with fold changes up to 4000 during *Candida* infection [132], suggesting lncRNAs may play a regulatory role in fungal infection. Despite little being known about lncRNA effects in the host response during *Candida* infection, ample evidence showed that lncRNAs are involved in the infection of bacteria and viruses [133]. To our knowledge, similar immune response might be induced during bacterial, viral, or fungal infections. On this basis, we assume that the lncRNAs might also participate in the anti-*Candida* host response, thus possessing the potential for specific targeting. 

### 4.1. LncRNAs Involved in the Host Defense

#### 4.1.1. NF-κB Signal Transduction Is Regulated by LncRNAs

**Figure 3:**
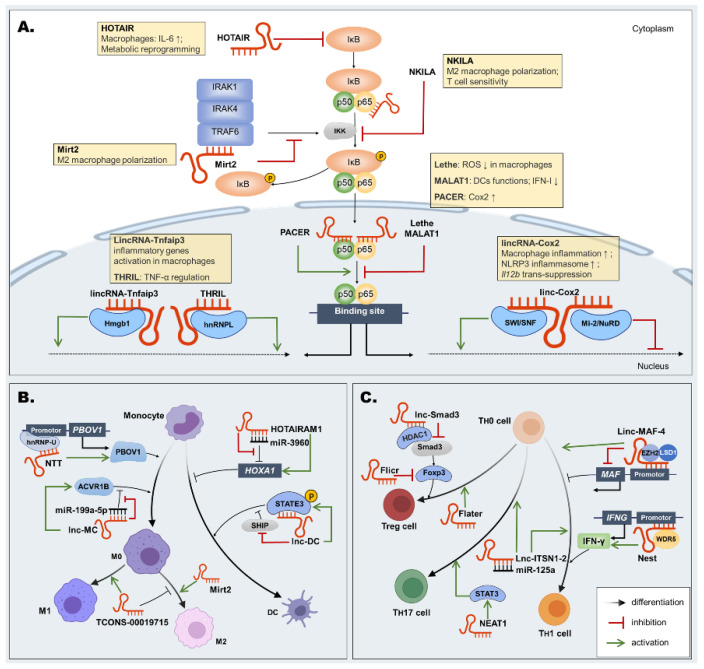
LncRNAs regulate NF-κB signal transduction, immune cell differentiation, and activation. (**A**) In the cytoplasm, HOTAIR facilitated the degradation of IκBα to enhance NF-κB signaling, while NKILA and Mirt2 inhibited the phosphorylation and degradation of IκB, thus suppressing NF-κB signaling. In the nucleus, PACER promoted the NF-κB DNA-binding ability. In contrast, Lethe and MALAT1 bound to p65 or p50/p65 to inhibit the DNA-binding ability of NF-κB. LincRNA-Cox2, lincRNA-Tnfaip3, and THRIL regulated the NF-κB-mediated target gene. LincRNA-Cox2 also inhibited the transcription of NF-κB target gene Il12b. (**B**) *PBOV1* and ACVR1B could facilitate monocyte–macrophage differentiation. lncRNA NTT promoted *PBOV1* transcription, and lnc-MC decreased ACVR1B degradation. TCONS_00019715 promoted M1 polarization, while Mirt2 promoted M2 polarization. STAT3 and *HOXA1* are essential for monocyte–DC differentiation, which were regulated by Lnc-DC and HOTAIRM1, respectively. (**C**) Linc-MAF-4 and NeST promoted differentiation of the T_H_1 cell, and Linc-MAF-4 inhibited T_H_1 differentiation. Lnc-ITSN1-2 contributed to T_H_1/T_H_17 cell differentiation. NEAT1 promoted T_H_17 cell differentiation by decreasing STAT3 degradation. *Foxp3* is very important for Treg differentiation. Flicr and Lnc-Smad3 inhibited the differentiation to Treg cells by inhibiting *Foxp3*. In contrast, Flater promoted Treg cell differentiation. HOTAIR, HOX transcriptional antisense RNA; IκBα, I kappa-B alpha; NKILA, NF-κB interacting lncRNA; Mirt2, myocardial infarction-related transcription factors 2; PACER, p50-associated cyclooxygenase-2 extragenic RNA; MALAT1, metastasis-associated lung adenocarcinoma transcript 1; LincRNA-Cox2, long intergenic noncoding RNA COX2; Tnfaip3, tumor necrosis factor α-induced protein 3; THRIL, heterogeneous nuclear ribonucleoprotein L immune-regulatory lncRNA; SWI/SNF, The switch/sucrose non-fermentable; hnRNPL, Heterogeneous nuclear ribonucleoprotein L; Mi-2/NuRD, Mi-2/nucleosomal remodeling and deacetylase; NTT, lncRNA noncoding transcript in T cells; hnRNP-U, the heterogenous ribonucleoprotein U; Lnc-MC, long noncoding monocytic RNA; ACVR1B: activin A receptor type 1B; Lnc-DC, long noncoding RNA DC; HOTAIRM1, HOXA transcript antisense RNA myeloid-specific 1; STAT3, signal transducer and activator of transcription 3; EZH2, enhancer of Zeste Homolog 2; LSD1, The lysine-specific demethylase; *IFNG*, Interferon-gamma gene; WDR5, WD repeat-containing protein 5; Flicr, *Foxp3* long intergenic noncoding RNA; *Foxp3*, Forkhead box P3; Lnc-Smad3; HDAC1, histone deacetylase 1; Flatr, *Foxp3*-specific lncRNA anticipatory of Tregs; Lnc-ITSN1-2, lncRNA ITSN1-2.

NF-κB proteins are a family of transcription factors that are of central importance in immunity, including P65 and p50. They can bind to a NF-κB inhibitor protein (IκB) in the cytoplasm. Under the catalysis of NF-κB kinase (IKK) enzymes, IκB is induced to release p65 and p50. P65 and p50 translocate into the nucleus and bind to specific binding sites to initiate the transcriptional process of inflammatory genes [134]. New evidence found 808 mRNAs and 51 lncRNAs differently expressed both in β-glucan-stimulated CD14^+^ monocytes and THP-1 cells, and they are involved in the NF-κB-signaling pathway [135]. We concluded that lncRNAs are likely to suppress the inflammatory response by interfering with the NF-κB signal pathways. The subcellular localization of lncRNAs partly determines the functions and modes of action [136]. Some of them are active in the cytoplasm, including NF-κB-interacting lncRNA (NKILA), myocardial infarction-related transcription factors 2 (Mirt2), and HOX transcriptional antisense RNA (HOTAIR), while others regulate in the nucleus, such as Lethe, metastasis-associated lung adenocarcinoma transcript 1 (MALAT1), and p50-associated cyclooxygenase-2 extragenic RNA (PACER) (Figure 3A). 

NKILA was reported to have inhibitory roles on NF-κB in HIV and asthma. NKILA affected HIV-1 replication and latency by suppressing the activity of HIV-1 long terminal repeat promoter in an NF-κB-dependent manner [137]. Interestingly, a novel lncRNA AK130181 also contributed to HIV-1 latency by regulating the promoter gene [138]. However, the regulating mechanism of AK130181 for inhibiting NF-κB is still unknown. In asthma, NKILA could limit airway inflammation by promoting M2 macrophage polarization and inhibiting the NF-κB pathway [139]. Mechanistically, in the cytoplasm, NKILA could bind to p65/IκB directly after stimulation by TNF-α or lipopolysaccharide (LPS), then cover the phosphorylation site of IκB, thereby inhibiting the phosphorylation and dissociation of IκB and the activation of the NF-κB pathway [140]. LncRNA NKILA was found to regulate the sensitivity of T cells to activation-induced cell death by inhibiting NF-κB activity [141]. These findings exemplified NKILA, widely participating in host immunity via inhibiting the NF-κB pathway, and is a potential therapeutic target for anti-*Candida* treatment. LncRNA Mirt2 in the cytoplasm also inhibited the NF-κB pathway. Mirt2, an LPS-induced lncRNA in macrophages, has been reported to regulate the NF-κB pathway by specifically inhibiting the ubiquitination of TRAF6, thus leading to a restraint of inflammatory responses after TLR4 activation. Mirt2 could also reduce inflammation by regulating the induction of macrophage polarization to the M2 phenotype [142,143], indicating that lncRNA Mirt2 could reduce macrophage mediated-inflammation by the NF-κB pathway. Conversely, lncRNA HOTAIR in the cytoplasm contributes to activation of the NF-κB pathway. Obaid et al. demonstrated that HOTAIR played key roles in LPS-induced metabolic programming in macrophages by activating NF-kB [144]. Besides, HOTAIR was significantly upregulated during hepatitis B virus (HBV) infection and may promote virus transcription and replication by elevating the activities of HBV promoters [145]. Mechanistically, HOTAIR could facilitate the degradation of IκBα, an inhibitory molecule of the NF-κB complex, to induce the expression of proinflammatory genes such as IL-6 in macrophages [146]. HOTAIR could also upregulate the TNF-α levels by modifying the p65 subunit at the posttranslational level [147]. Interestingly, in HCV-4 patients, HOTAIR could serve as a risk assessment biomarker following direct-acting antiviral therapy [148], suggesting that HOTAIR has potential as a biomarker for the treatment or risk evaluation of infectious diseases.

In the nucleus, Rapicavoli et al. reported that lncRNA Lethe might inhibit the DNA-binding activity of NF-κB through its interactions with p65 [149]. Lethe could be selectively induced by proinflammatory cytokines TNF-α or IL-1β in mouse embryonic fibroblasts [149] or be upregulated by activated STAT3, then promote HCV replication through a negative regulatory mechanism of the type I IFN response [150]. Lethe also regulated the production of ROS in macrophages through modulating NOX2 gene expression via NF-κB signaling [151]. Thus, Lethe could play an anti-inflammatory role by acting directly on NF-κB proteins. MALAT1 was also demonstrated as a negative intranuclear regulator of NF-κB. MALAT1 expressed in LPS-induced macrophages could suppress the NF-κB DNA-binding activity by physically interacting with both p65 and p50 [152]. MALAT1 has also been reported to regulate the function of DCs through NLRP3 as a competing endogenous RNA (ceRNA) [153]. The altered expression of MALAT1 was found both in viral infection and sepsis [154,155]. MALAT1 seems to be more involved in viral infection, such as HPV [156], enterovirus type 71 [157], flavivirus [158], and SARS-CoV-2 [157]. Mechanistically, MALAT1 acted as a negative regulator of IFN-I production to suppress antiviral innate responses via the RNA–RBP interactive network [154]. MALAT1 also acted as a promoter of HIV-1 transcription to promote HIV-1 transcription and infection at an epigenetic level [159]. However, MALAT1 was not necessary in the T-cell-mediated antiviral response [160]. Little lncRNAs in the nucleus could contribute to activate the NF-κB pathway. Krawczyk et al. illustrated that lncRNA PACER could combine with the inhibitory subunit p50, blocking the formation of p50/p50. Then, the activated dimer p50/p65 NF-κB bound to the promoter of Cox2 to increase the transcription of Cox2 [161]. In summary, lncRNAs may be promising molecules for regulating NF-κB signaling by directly targeting pathway elements.

The upstream and downstream genes of the NF-κB pathway could also be regulated by lncRNAs. These lncRNAs include tumor necrosis factor-alpha and heterogeneous nuclear ribonucleoprotein L-related immunoregulatory lincRNA (THRIL), lincRNA-Tnfaip3, long intergenic noncoding RNA COX2 (lincRNA-Cox2), etc. In the priming phase of the immune response, the classic signaling mode is the NF-κB family of transcription factors in response to TNF. LncRNA THRIL located next to the *Bri3bp* gene may interact with the binding proteins hnRNPL in the promoter region of the *TNF* gene cluster and form a functional THRIL–hnRNPL complex, which is essential for the transcription of TNF-α [162,163]. THRIL could also inhibit the expression of TNF-α by interacting with microRNAs (miRNAs) [164]. Additionally, lincRNA-Tnfaip3 has also been reported to mediate the inflammatory genes regulated by NF-κB. LincRNA-Tnfaip3 could interact with high-mobility group box 1 (Hmgb1) to assemble a NF-κB/Hmgb1/lincRNA-Tnfaip3 complex, which transactivated NF-κB-mediated inflammation in macrophages [165]. LincRNA-Cox2 is an early NF-ĸB responsive gene triggered by TNF-α stimulation [166]. The inflammatory response in macrophages infected by *Mycobacterium tuberculosis* and *Listeria monocytogenes* was also modulated by lincRNA-Cox2 via the activation of NF-κB [167,168]. Mechanistically, Hu et al. found that lincRNA-Cox2 reacted with the subunit of NF-κB [169]. LincRNA-Cox2 has also been reported to activate NLRP3 inflammasome and autophagy by binding p65 and promoting its nuclear translocation and transcription [170]. LincRNA-Cox2 also regulates the NF-ĸB responsive gene as negative feedback, such as *Il12b*. The *Il12b* gene is characterized by a delayed transcription of the gene following NF-ĸB signaling activation. LincRNA-Cox2 assembled into the Mi-2/NuRD complex and recruited to the *Il12b* gene locus, resulting in trans-suppression through histone modification [166]. These findings suggested that lincRNA-Cox2 mediated a negative feedback loop activated by the NF-κB pathway; that is, lincRNA-Cox2 was activated by the NF-ĸB pathway and then restricted the overexpression of the NF-ĸB-responsive proinflammatory cytokine. On this basis, targeting lncRNAs or co-targeting lncRNAs and NF-κB appear to be a promising strategy.

#### 4.1.2. LncRNAs Participate in Immune Cell Differentiation and Activation

As mentioned above, monocytes and T_H_ cells can evolve into several distinct cell types. Ample evidence has concluded that lncRNAs may regulate the differentiation and activation of monocytes and T_H_ cells [171], including noncoding transcripts in T cells (NTT), long noncoding monocytic RNA (lnc-MC), TCONS_00019715, lincRNA-Cox2, long noncoding RNA DC (lnc-DC), the HOXA transcript antisense RNA myeloid-specific 1 (HOTAIRM1), NeST, Linc-MAF-4, *Foxp3*-specific lncRNA anticipatory of Tregs (Flatr), *Foxp3* long intergenic noncoding RNA (Flicr), Lnc-Smad3, etc. (Figure 3B,C).

Macrophages are developmentally derived from circulating monocytes, and their phenotype transformation can be modulated by lncRNAs. For example, lncRNA NTT led to cell cycle G1 arrest in monocytes by binding hnRNP-U to the promoter of the adjacent gene *PBOV1*, which could facilitate differentiation into macrophages [172]. In addition, Xie et al. determined that lnc-MC was upregulated in monocytes and acted as a ceRNA to sponge and isolate miR-199a-5p, which reduced the inhibition of activin A receptor type 1B (ACVR1B) expression [173], suggesting lnc-MC could promote monocyte differentiation into macrophages. Moreover, Huang et al. found that a reduction in TCONS_00019715 expression could promote the transition of macrophages from the M1 phenotype to the M2 phenotype [174]. In a septic mouse model, overexpressing lincRNA-Cox2 enhanced the effect of LPS on inflammation and macrophage polarization, while silencing lincRNA-Cox2 declined the percentage of M1 macrophages and increased the percentage of M2 macrophages [175]. Monocytes also differentiate into DCs. lnc-DC is a type of lncRNA exclusively and highly expressed on DCs. Genes related to DCs function were downregulated, while monocyte marker CD14 was upregulated with the knockdown of lnc-DC expression during monocyte–DC differentiation [176]. Functionally, lnc-DC could prevent the dephosphorylation of STAT3 in the cytoplasm, which may be important for the differentiation of monocytes to DCs [177]. Lnc-DC could also regulate proinflammatory cytokines stimulated by LPS or hepatitis B virus (HBV) [178]. Furthermore, lncRNA HOTAIRM1 is a negative regulator of monocyte–DC differentiation. Xin et al. showed that HOTAIRM1 was able to pair competitively with miR-3960 as a ceRNA, while miR-3960 could promote DCs differentiation by inhibiting *HOXA1*. When HOTAIRM1 was knocked down, monocyte markers CD14 and B7H2 were downregulated [179].

The T_H_ cell effector subsets include T_H_1 cells, T_H_2 cells, T_H_17 cells, and Treg cells. The differentiation and expansion of these subset cells contribute to the inflammatory response and host defense. IFN-γ has a direct effect resulting in differentiation to T_H_1 cells and inhibition of differentiation to T_H_2 cells. LncRNA NeST and Linc-MAF-4 were found to be related with T_H_1 cell and T_H_2 cell differentiation. LncRNA NeST could bind to WDR5 and form a transcriptional activation complex. The complex induced *IFNG* transcription in NK cells, which was reported to enhance the sensitivity of mice to Theiler’s virus infection and resistance to *Salmonella typhimurium* infection [180,181,182]. Recent studies have demonstrated that NeST contributed to the immune response during *brucellosis* and *leishmania braziliensis* infection through a positive role in enhancing INF-γ expression [183,184]. NeST and Linc-MAF-4 were upregulated in acute leukemia patients after transplantation [185], suggesting they might act as a suitable prognostic indicator. In addition to IFN-γ, Linc-MAF-4 also promoted the differentiation of T_H_0 cells into T_H_1 cells by inhibiting the transcription of T_H_2-associated transcription factor *MAF*. Linc-MAF-4 acted as a scaffold to suppress the activation of the *MAF* promoter, thereby depressing *MAF* transcription and T_H_2 differentiation [186]. T_H_17 responses are relevant to preventing pathogenic infections by the commensal fungus *Candida*. It has been reported in inflammatory bowel disease that Lnc-ITSN1-2 promoted T_H_1/T_H_17 cell differentiation by acting as a ceRNA for IL-23R through sponging miR-125a [187]. LncRNA NEAT1 played a similar role as an auxo-active molecule in promoting T_H_17 cell differentiation. NEAT1 reduced the ubiquitination level of its downstream molecule STAT3, which was a critical molecule for T_H_17 cell differentiation [188]. Interestingly, NEAT1 was also involved in T_H_2 cell activation and macrophage polarization. NEAT1 upregulated STAT6 via inhibiting its ubiquitination, thereby promoting T_H_2 cell activation [189]. NEAT1 modulated the macrophage function via promoting M2 polarization induced by LPS or suppressing apoptosis during mycobacterium tuberculosis infection, acting as a ceRNA [190,191]. Emerging evidence suggests NEAT1 was associated with periodontitis and several virus infections, such as Hantavirus, herpes simplex virus-1, HIV, etc. [192,193,194,195]. Forkhead box P3 (*Foxp3*) is a key transcriptional regulator of Tregs. LncRNA Flatr was speculated to enhance the immunosuppressive function of Treg cells by promoting the expression of *Foxp3* [196]. On the contrary, lncRNA Flicr inhibited the expression of *Foxp3* to suppress Treg cells by modifying the chromatin accessibility [197]. In addition, Lnc-Smad3 could interact with histone deacetylase histone deacetylase 1 and silence the transcription of Smad3, which mediated *Foxp3*-induced Treg cell polarization [198]. In sum, the lncRNAs mentioned in this part seem to have a higher cell specificity, which shows in their naming and functions. These cell-specific lncRNAs might be used as new targets for cellular immunotherapy.

#### 4.1.3. Other LncRNAs in the Host May Respond to *Candida*

Some lncRNAs may be associated with the anti-*Candida* response. However, the molecular mode is not mediated by NF-κB signaling transduction or immune cell differentiation or activation. LncRNAs could be regulators of cytokine secretion, such as Lnc-IL-7R, lncRNA IL7-AS, AS-IL1α, IL1β-eRNA, IL1β-RBT46, LncBST2/BISPR, lncISG15, NRAV, and ROCKI. Lnc-IL-7R was upregulated in response to TLR2/TLR4 agonists and inhibited inflammation by reducing the expression of IL-6 and IL-8. Similarly, LPS-induced lncRNA IL7-AS in macrophages led to the downregulation of IL-6 and exerted proinflammatory activity [199]. LncRNA AS-IL1α has been identified as an important regulator of IL-1α transcription in macrophages during the innate immune response [200]. LncRNA IL1β-eRNA and IL1β-RBT46 were nuclear-located transcripts, located surrounding the *IL1β* locus and modulated by NF-κB. They seem to regulate *IL1β* transcription in cis given the genomic position, while they also promote *CXCL8* transcription in trans in human monocytes stimulated by LPS [201]. Moreover, some lncRNAs regulate the levels of many IFN-stimulated genes. LncBST2/BISPR and lncISG15 regulated the levels of many IFN-stimulated genes, which may act as potent antivirals [202]. Conversely, another lncRNA, NRAV, downregulated after infected influenza A virus, worked as a negative regulator of IFN-stimulated genes [203]. Additionally, Zhang et al. observed the link between the activity of lncRNA ROCKI, which was involved in TLR signaling with an unknown function, and the risk of inflammation and infection-related disease phenotypes [204]. These suggested an important regulating role for lncRNAs in the human immune response, paving new avenues for cytokine therapy. 

There are other directions that traditional anti-*Candida* HDTs do not target, including the skin and mucosa barrier, NLRP3 inflammasome response, and ceRNA mechanisms. LncRNAs can play antifungal roles by regulating the barrier function of skin and mucosa. For instance, a recent study revealed the antifungal effect of lncRNA 9708-1 in a vulvovaginal candidiasis murine model. LncRNA 9708-1 was identified to play a protective role by upregulating the expression level of FAK, which was expressed mainly in the epithelial basal layer [205]. The skin-specific lncRNA TINCR played a role in the induction of key protein mediators involved in epidermal barrier formation [206]. LncRNA H19 was also reported to regulate the intestinal mucosal mechanical barrier in hindering the invasion pathogen [109], and lncRNA ANRIL might act as potential therapeutic targets for Crohn’s colitis due to its promoting the proliferation of epithelial cells and reducing apoptosis [207]. Thus, targeting TINCR, H19, and ANRIL can be used to enhance the first barrier of the host defense. 

Recent studies have also emphasized the function of lncRNAs in the control and regulatory activity of the NLRP3 inflammasome, including lncRNA NEAT1, MALAT1, lincRNA-EPS, and 1810058I24Rik. NEAT1 was associated with the NLRP3, NLRC4, and AIM2 inflammasomes to enhance their assembly and stabilize caspase-1 to promote IL-1β production [208]. However, Wang et al. reported that NEAT1 may induce autophagy and suppress the NLRP3 inflammasome to alleviate LPS-induced inflammation [209]. NEAT1 in macrophages played a proinflammatory role while inhibiting inflammation in osteosarcoma cells, suggesting that the specific target cell type needs to be considered when targeting NEAT1. Furthermore, MALAT1 could promote the NLRP3 inflammasome expression as sponges of miR-133 or miR-203 [210]. MALAT1 has also been proven to represent a potential therapeutic target for inhibiting breast cancer progression [211]. LincRNA-EPS has been shown to negatively regulate activation of the NLRP3 inflammasome by suppressing the expression of the ASC adaptor protein [212]. Bhatta et al. found that lncRNA 1810058I24Rik in the cytosol promoted the translation of a 47 amino acid peptide named mitochondrial micropeptide-47 responsible for the NLRP3 inflammasome response [213]. Therefore, the lncRNA-induced NLRP3 inflammasome response is also one of the potential targets of immunotherapy in infectious diseases. 

In addition, most of studies on lncRNAs are explained by ceRNA mechanisms, which means lncRNAs acting as ceRNAs may hijack miRNAs to inhibit their functions. miRNAs are small endogenous noncoding RNAs capable of inhibiting or degrading the target mRNA through complementary base paring at the post-transcriptional level [214]. Since ample studies have explored the influence of miRNAs following *Candida* exposure [215], lncRNAs may ultimately play an opposite regulatory function via miRNA sponging mechanisms. For instance, miR-129 could respond to *Candida* infection in DCs [216]. MAP3K7 was an activator of the NF-κB pathway in multiple myeloma and could be targeted by miR-129. LncRNA PCAT-1 regulated the cell cycle by sponging miR-129 to activate the MAP3K7/NF-κB-signaling pathway [217]. In addition, miR-155, miR-146a, miR-125a, and miR-455 could be upregulated by NF-κB and most likely induced by LPS or by heat-killed *Candida* via TLR4 or dectin-1 [218]. LncRNA CYTOR, HCG18, NEAT1, and SOX2-OT served as sponges of miR-155, miR-146a, miR-125a, and miR-455-3p, respectively [190,219,220,221]. Among them, lncRNA CYTOR as a ceRNA for miR-155 was proven to counteract the miR-155-induced inhibiting effect of IKBKE, thereby inhibiting the NF-κB pathway in pathological cardiac hypertrophy [221]. Thus, we presume that these lncRNAs might mediate the host immune responses against *Candida* through targeting miRNAs; however, the exact function and mechanism during *Candida* infection remain to be elucidated.

In summary, lncRNAs are actively involved in the NF-κB-signaling pathway, immune cell activation, regulation of cytokines, the ceRNA network, etc. Of note, the lncRNAs involved in the host response listed in Table 2 were not solely from studies of *Candida* exposure, given the lack of associated research. LncRNAs in the host response possess anti-*Candida* potency and a promising future in HDTs. Next, there is a problem to be solved as to whether lncRNAs could be developed as therapeutic targets.

### 4.2. Advantages and Feasibility of LncRNAs in HDTs

Discoveries in lncRNA biology have aroused interest in probing the transcriptome profiles of different diseases. In the field of botany, the regulatory roles of lncRNAs in the occurrence and development of mycosis have been disclosed [222,223]. Strategies that utilize lncRNAs such as RNA interference (RNAi) to develop the resistance of plants to fungi are being studied as well [222,224,225]. In human diseases, lncRNAs have been identified to play critical regulatory roles in diverse biological process, which afford legible targetable key sites for subsequent modulating. Clinical trials based on nucleic therapies are also being conducted [226]. Different from other RNA molecules, lncRNAs exhibit highly specific expression patterns in the cell, organ, and spatiotemporal distribution [227]. Furthermore, another possible advantage of targeting lncRNAs is their relatively lower expression levels compared with mRNAs; that is, a slight number of lncRNAs may also show regulating functions [228]. These features make lncRNAs more specific and promising therapeutic targets.

The current prevalent approaches to targeting lncRNAs are mainly categorized into three groups: targeting lncRNA directly, targeting the lncRNA-expressing loci, or interfering with the secondary or tertiary structures of lncRNAs [229]. Among them, it is arguably the most popular methods to target lncRNAs directly, including double-stranded RNAi and single-stranded antisense oligonucleotides (ASOs). The RNAi therapy, based on small interfering RNAs (siRNAs), targets lncRNA complementarily to their nucleic acid sequence and then recruits the RNA-induced silencing complex to induce lncRNA degradation. The therapy can achieve significant effect in various diseases due to the high selectivity and knockdown efficiency for RNA. For example, in a triple-negative breast cancer nude mice model, DANCR siRNA nanoparticles were systematically administered and suppressed tumor progression with no obvious side effects [230]. Compared to siRNA, ASOs were shown to be more efficient in targeting nuclear lncRNAs. ASOs, as synthesized, short single-stranded oligonucleotides, could specifically pair to their target lncRNAs, thus degrading their transcripts. For example, LNA gapmeR was a type of ASO designed specifically for lncRNA and mRNA. Amodio et al. reported that the delivery of MALAT1-targeting 16mer LNA gapmeR g#5 enhanced the antitumor activity in a humanized murine model of multiple myeloma [231]. Notably, to date, more than 20 nucleic therapies have been approved for marketing. Patisiran is the first commercial siRNA-based drug that was approved for the treatment of hereditary amyloidogenic transthyretin amyloidosis with polyneuropathy in adults in 2018 [232]. A siRNA-based therapeutic Inclisiran, directed against PCSK9 mRNA, was approved in the European Union in 2020, using in adults with primary hypercholesterolemia or mixed dyslipidemia [233]. The application of ASO-based drugs, such as eteplirsen and nusinersen, was mainly focused on the field of spinal muscular atrophy and Duchenne muscular dystrophy [234,235]. Recently, the FDA approved a phase I clinical trial of Andes-1537, an ASO that targeted mitochondrial lncRNAs (NCT02508442 and NCT03985072). It was reported that Andes-1537 was well-tolerated for patients with advanced solid tumors [236]. 

However, the major drawbacks of nucleic therapeutics are instability and low access, due to the characteristics easy to hydrolyze and the location of the RNAs. The core issue of the challenges is the optimization of the delivery system. An efficient delivering system must satisfy the requirements of stability and cell permeability. The delivery system can be divided into exogenous and endogenous vectors. Exogenous vectors include the classical viral vectors like lentiviral vectors or adenoviral vectors and nonviral vectors such as liposomes (NPs) and lipid NPs. Viral vectors have been used in laboratories for the stable and efficient interference of lncRNAs. However, their safety hazard means that they are substituted by other vectors in clinical settings. Research about NPs has achieved significant progress due to the safety and nanomaterial development [237]. Lipid nanoparticles (LNPs) can be easily modified and are more suitable for encapsulating several nucleic acid drugs. For instance, patisiran was delivered by LNP. In the lncRNA field, LNPs have also achieved ideal results in animal models. The ASO-Au-TAT NPs targeting MALAT1 inhibited cancer metastasis, and the RGD-PEG-ECO/siDANCR nanoparticles could suppress tumor progression [230,238]. Moreover, endogenous exosomes were developed to achieve a lower immunogenicity and better biocompatibility [239]. LncRNA-H19 delivered by high-yielding extracellular vesicle–mimetic nanovesicles exhibited the ability to accelerate wounds healing in a diabetic rat model [240]. Due to the consistent progress of nucleic acid delivery systems, novel therapies targeting lncRNAs are becoming a reality in clinical settings.

## 5. Conclusions and Future Perspectives

**Figure 4:**
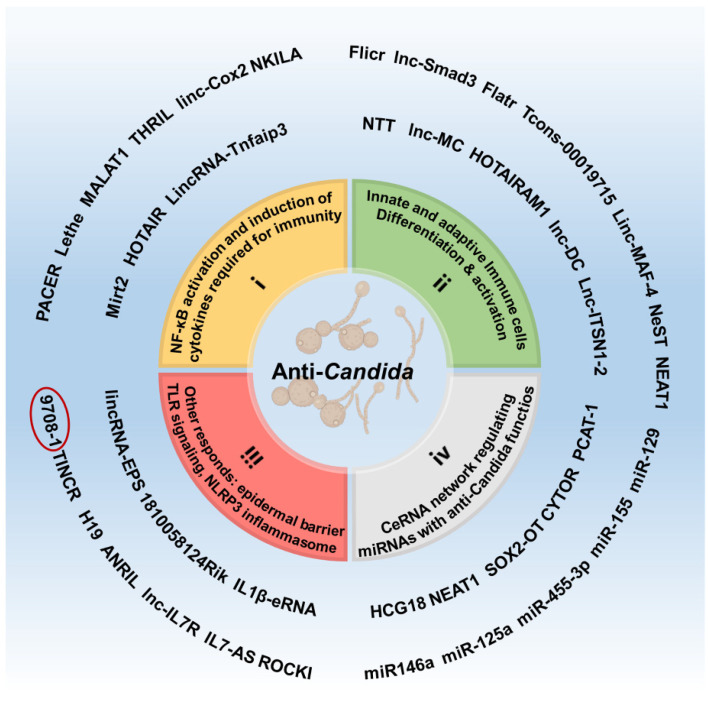
Hypothetical role of lncRNAs as HDT targets against *Candida*. LncRNAs have the potential to act as regulators in host immunity by regulating (i) the NF-κB pathway, which contributes to downstream cytokines involved in the anti-*Candida* immunity; (ii) differentiation and activation of crucial innate or adaptive immune cells; (iii) controlling the cytokine-related pathway, the epidermal barrier, or activation of the NLRP3 inflammasome; and (iv) anti-*Candida* miRNA via the ceRNA network. Red circle: lncRNA 9708-1 has been shown to be involved in response to candidiasis [205].

In the present review, we concluded that HDTs based on the *Candida*–host interaction exerted a therapeutic role in candidiasis. In addition to cytokine and immune cell targets that have been used, it is feasible to target the related lncRNAs to modulate the host immune defense during infection. LncRNAs related to NF-κB signaling, immune cell activation, cytokine regulation, the ceRNA network, etc. were predicted to have great potential for anti-*Candida* therapy (Figure 4). Clinical trials om the lncRNA field have also been performed and show that lncRNAs have a promising future in targeted therapy. The possible application strategy focuses on two aspects: selected targets should amplify the sensing signals and intensify the cascade reaction, thus activating the inflammatory pathway and recruiting the immune cells, or should modulate the inflammation response to achieve a balance between pathogen clearance and organ damage. Undoubtedly, it will become indispensable to combine precision medicine with traditional pathogen-directed agents, while the HDTs may provide a more personalized approach adjusted for the patient. However, the lncRNAs-based strategy for candidiasis is still in infancy and requires more in-depth insight into their biological outcomes and mechanisms. 

## Figures and Tables

**Table 1:** Eleven clinical trials of host-directed therapies.

Author (Year)	Research Type	Subjects (M/F)	Diseases	Study Design	Outcome	Clinical Trials
Levy et al., 1989 [124]	Prospective study	22^P^ F	Vulvovaginal candidiasis	D.651 vaccine with different doses	Reduced recurrence rate	Phase II
Nemunaitis et al., 1991 [125]	Prospective study	24^T^ (17M, 7F)	Invasive *Candida* infection after transplantation	rhM-CSF with dose escalation	Resolution of fungal infection	Phase I
Nemunaitis et al., 1993 [126]	Prospective study	46^T^ (28M, 18F) 58^P^ (34M, 24F)	Invasive *Candida* infection after transplantation	rhM-CSF + amphotericin	Reduced recurrence rate	
Vazquez et al., 2000 [102]	Prospective study	11^T^ (10M, 1F)	AIDS with fluconazole-refractory oropharyngeal candidiasis	rh-GM-CSF + fluconazole	Clinically improved and colony counts decreased	
Pachl et al., 2006 [116]	RCT	56^T^ (42M, 14F) 61^P^ (35M, 26F)	Invasive candidiasis	Mycograb/Placebo + amphotericin B	Quicker clearance of the infection and reduced rate of mortality	
Schmidt et al., 2012 [114]	RCT	15^T^ 15^T^ 10^P^	Healthy adults	30 μg NDV-3 300 μg NDV-3 Placebo	Induced rapid and robust anti-Als3p IgG and IgA1 responses and a substantial T-cell response	Phase I
Takeuchi et al., 2014 [120]	RCT	13^T^ (2M, 11F) 13^P^ (3M, 10F)	Older people	Anti-CA IgY Placebo	Reduced number of *Candida* CFU	
Delsing et al., 2014 [96]	Prospective study	8^T^ (5M, 3F) 3^P^ F	Invasive fungal infection	rIFN-γ/Placebo + Antifungals	Restored immune function including increased HLA-DR expression and an enhanced capacity of leukocytes	
Wan et al., 2015 [121]	RCT	68^T^ (51M, 17F) 69^T^ (44M, 25F) 69^T^ (37M, 32F)	Allogeneic hematopoietic stem-cell transplantation	GM-CSF G-CSF GM-CSF + G-CSF	Lower invasive fungal disease-related mortality	Phase IV
Edwards et al., 2018 [122]	RCT	67^T^ F 12^T^ F 64^P^ F	Recurrent vulvovaginal candidiasis	NDV-3A/NDV-3/Placebo + fluconazole	Safe and highly immunogenic and reduced recurrent frequency	Phase II
Xie et al., 2019 [123]	RCT	208^T^ (135M, 73F) 208^P^ (128M, 80F)	Children in the pediatric intensive care units	Vitamin D Placebo	Reduced infections of *Candida*	

^T^, treatment group; ^P^, placebo group; M, Male; F, Female; RCT, Randomized Controlled Trial; D.651, a vaccine (including the ribosomes of *Candida* albicans serotypes a and b); rhM-CSF, recombinant human macrophage colony-stimulating factor; rh-GM-CSF, recombinant human granulocyte macrophage-colony stimulating factor; GM-CSF, granulocyte macrophage-colony stimulating factor; Mycograb, a human recombinant monoclonal antibody against heat shock protein 90; NDV-3, an immunotherapeutic vaccine (consisting of an adhesin/invasin from *Candida* albicans and extraneous sequences); anti-CA IgY, an egg yolk antibody against *Candida* albicans; rIFN-γ, recombinant interferon-gamma; G-CSF, granulocyte colony-stimulating factor; NDV-3A, an immunotherapeutic vaccine (containing an adhesin/invasin from *Candida* albicans without extraneous sequences).

**Table 2:** The lncRNAs involved in the host response.

LncRNA	Expression	Functions	Mechanisms	References
NKILA	Breast cancer cell	Inhibit NF-κB	Inhibit IκB degradation	[140]
Mirt2	Macrophage	Inhibit NF-κB	Inhibit IKK phosphorylation	[142,143]
Lethe	Fibroblast	Inhibit NF-κB	Interact with p65	[149]
MALAT1	Macrophage; DC	Inhibit NF-κB	Interact with p50 and p65	[152,153]
HOTAIR	Macrophage; Fibroblast	Activate NF-κB	Facilitate IκBα degradation	[146,147]
PACER	Monocyte; Macrophage	Activate NF-κB	Promote p50/p65 dimer formation	[161]
lincRNA-Cox2	Macrophage	Activate NF-κB	Recruite SWI/SNF or Mi-2/NuRD	[166]
THRIL	Macrophage	Activate NF-κB	Interact with hnRNP-L	[162,163]
lincRNA-Tnfaip3	Macrophage	Activate NF-κB	Assemble NF-κB/Hmgb1/lincRNA-Tnfaip3 complex	[165]
NTT	Monocyte	Promote macrophage differentiation	Promote *PBOV1* transcription	[172]
lnc-MC	Monocyte	Promote macrophage differentiation	Activate ACVR1B	[173]
lnc-DC	DC	Promote DC differentiation	Promote STAT3 activation	[176]
HOTAIRM1	Monocyte; DC	Inhibit DC differentiation	Activate *HOXA1*	[179]
NeST	T cell; NK cell	Promote T_H_1 cell differentiation	Promote *IFNG* transcription	[180]
linc-MAF-4	T_H_ cell	Inhibit T_H_1 cell differentiation	Depress *MAF* expression	[186]
NEAT1	T_H_ cell	Promote T_H_17 cell differentiation	Reduce STAT3 degradation	[188]
Flicr	Treg cell	Inhibit Treg cell differentiation	Inhibit *Foxp3*	[197]
lnc-Smad3	Treg cell	Inhibit Treg cell differentiation	Inhibit Smad3	[198]

## Data Availability

Not applicable.
